# Feasibility of Virtual Tablet-Based Group Exercise Among Older Adults in Siberia: Findings From Two Pilot Trials

**DOI:** 10.2196/mhealth.7531

**Published:** 2018-02-27

**Authors:** Svetlana Nikitina, Daniele Didino, Marcos Baez, Fabio Casati

**Affiliations:** ^1^ Tomsk Polytechnic University Tomsk Russian Federation; ^2^ Department of Information Engineering and Computer Science University of Trento Trento Italy; ^3^ Department of Psychology Humboldt-Universität zu Berlin Berlin Germany

**Keywords:** physical fitness, exercise training, tablet computers, elderly, social support

## Abstract

**Background:**

Regular physical activity has a positive effect on physical health, well-being, and life satisfaction of older adults. However, engaging in regular physical activity can be challenging for the elderly population because of reduced mobility, low motivation, or lack of the proper infrastructures in their communities.

**Objective:**

The objective of this paper was to study the feasibility of home-based online group training—under different group cohesion settings—and its effects on adherence and well-being among Russian older adults. We focused particularly on the technology usability and usage and on the adherence to the training (in light of premeasures of social support, enjoyment of physical activity, and leg muscle strength). As a secondary objective, we also explored the effects of the technology-supported intervention on subjective well-being and loneliness.

**Methods:**

Two pilot trials were carried out exploring two different group cohesion settings (weak cohesion and strong cohesion) in the period from 2015 to 2016 in Tomsk, Russian Federation. A total of 44 older adults (59-83 years) participated in the two pilots and followed a strength and balance training program (Otago) for 8 weeks with the help of a tablet-based virtual gym app. Participants in each pilot were assigned to an interaction condition, representing the online group exercising, and an individual condition, representing a home-based individual training. Both conditions featured persuasion strategies but differed in the ability to socialize and train together.

**Results:**

Both interaction and individual groups reported a high usability of the technology. Trainees showed a high level of technology acceptance and, particularly, a high score in intention to future use (4.2-5.0 on a 5-point Likert scale). Private texting (short service message [SMS]) was used more than public texting, and the strong cohesion condition resulted in more messages per user. Joint participations to training sessions (copresence) were higher for the social group with higher cohesion. The overall adherence to the training was 74% (SD 27%). Higher levels of social support at baseline were associated with higher adherence in the low cohesion condition (F1,18=5.23, *P*=.03), whereas in the high cohesion, such association was not found. Overall improvement in the satisfaction with life score was observed between pre and post measures (F1,31=5.85, *P*=.02), but no decrease in loneliness.

**Conclusions:**

Online group exercising was proven feasible among healthy independently living older adults in Russia. The pilots suggest that a physical training performed in a virtual environment positively affect the life satisfaction of the trainees, but it does not provide support for a decrease in loneliness. High cohesion groups are preferable for group exercising, especially to mitigate effects of low social support on adherence. Further research in motivating group interactions in training settings is needed.

## Introduction

### Background

Regular physical activity is a key factor to a successful aging, contributing to positive outcomes in health and well-being in later life [1-4]. It can improve physical function [4], slow the progression of degenerative diseases [3], reduce risk of falls [1], and also improve cognitive performance, mood, and quality of life (QoL) of older adults [2,4]. A physically inactive lifestyle, on the contrary, can increase the risk of developing chronic diseases, one of the leading causes of death and disability in older adults [5,6].

Engaging in regular physical activity can be challenging. Older adults might suffer from reduced mobility, low self-efficacy, lack the proper infrastructures in their communities, or simply find it difficult to leave home and participate in physical activities on a regular basis [7,8]. For these and many other reasons, physical inactivity is still prevalent in older adults [9], leading to the undesired effects on health and well-being.

Intervention programs to promote physical activity have shown to be effective in increasing and maintaining physical activity [10]. In particular, group-based interventions have shown promising results in long-term settings with higher adherence compared with individual home-based interventions. Studies have also reported a preference by older adults for group exercising [11] and discussed the potential of the social context to stimulate social interactions and increase social well-being [12].

However, despite the body of literature on the topic, little attention has been paid on populations living under difficult environmental conditions and undergoing complex social changes, such as the Siberian community. Seasonal fluctuation has been found to determine the level of physical and social activities of older adults [13] leading to less opportunities to go out and interact, especially in high latitudes where winter can result in a decline of physical functions of older adults, such as ankle strength [14]. Recent history has also shaped the lives of older adults in Russia. The breakup of Soviet Union in the early 90s, and the difficult years that followed, negatively affected the social and economic well-being of the Russian population: the life expectancy of men is 14 years lower than in the European Union [15], and loneliness levels are among the highest in Europe [16]. The social, political, and economic uncertainty also deeply affected QoL, with a decrease in life satisfaction and happiness [17].

The above observations point to the need for solutions that can help older adults living under the above conditions to keep physically and socially active. Technology-supported interventions have been shown in the past to be successful in this goal [18].

### Related Work

Recent research has demonstrated an effectiveness of technology-supported exercise interventions for older adults in terms of physical fitness [18]. However, although there is an ongoing discussion on whether group exercising or home-based individual exercising is more effective in increasing adherence of individuals to training programs (eg, [19,20]) and despite calls for analysis focusing on understanding group-based exercising in terms of *cohesiveness* (frequency of contact and group dynamics) [21], no intervention has compared the effectiveness of individual and (different types of) group settings in a technology-supported intervention.

Research has also shown a preference by older adults in group training [11,12]. However, implementing group exercising can be challenging, especially in a heterogeneous elderly population, with individual differences leading to motivational issues and problems in tailoring the training [11].

Fitness apps for home-based training have been widely explored in technology-supported interventions (see [22] for a review); however, we are not aware of interventions supporting online group exercising for individuals of different levels of fitness. Consequently, there is very limited research on the effects of level of fitness, social support, and subjective well-being in online group settings. The exception comes from a recent study on an Internet-based group training intervention [23] relying on a general-purpose teleconference software to deliver real-time exercises to older adults in rural areas. Although targeting homogeneous groups, focused on physical fitness outcomes, and limited to a small sample of 10 older adults, the study highlights some interesting challenges in deploying this type of technology.

In our previous study [12,24], we made some steps to test the feasibility of a tool for online group exercising, namely Gymcentral, that allows individual of different levels of fitness to follow exercises with the remote company of others. We conducted an 8-week pilot study exploring the effects of online group exercise training in Trento, Italy, with 37 adults, 65 years and above, who followed the Otago exercise program [25] aiming at strength and balance improvement in older age. The specific focus of the study was on technology acceptance, attitude, and preference toward group training and its effects on physical and social well-being; in comparison with a traditional tablet-based individual training program implementing no persuasion strategies.

Still, despite the prior work and the extensive existing literature, open questions remain:

1. How does the online group exercising translate to other cultural and environmental settings?
2. How effective is online training with groups of different levels of cohesion?
3. How does online group exercising compare with individual training featuring persuasion strategies?

### Objectives

This paper reports on two pilot studies of an online exercise intervention with older adults living in Tomsk, Siberian Federal District (Russia). The aim of the intervention was to enable older adults of different levels of fitness to follow a personalized exercise program from home, with the (virtual) company of training companions and under the supervision of a remote coach. This was done with the support of a tablet app offering group exercising in a virtual gym while leveraging on the social context of the group exercising to enable social interactions and feedback.

The main objective of the pilot was to study the feasibility of online group exercising under different cohesion settings among Siberian older adults. We focused on the technology acceptance, on the adherence to the training (especially in light of pre-measures of social support, as well as on the enjoyment of physical activity and leg muscle strength). As a secondary objective, we also explored the effects of the technology-supported intervention on subjective well-being and loneliness.

## Methods

### Training Apps

**Figure 1:**
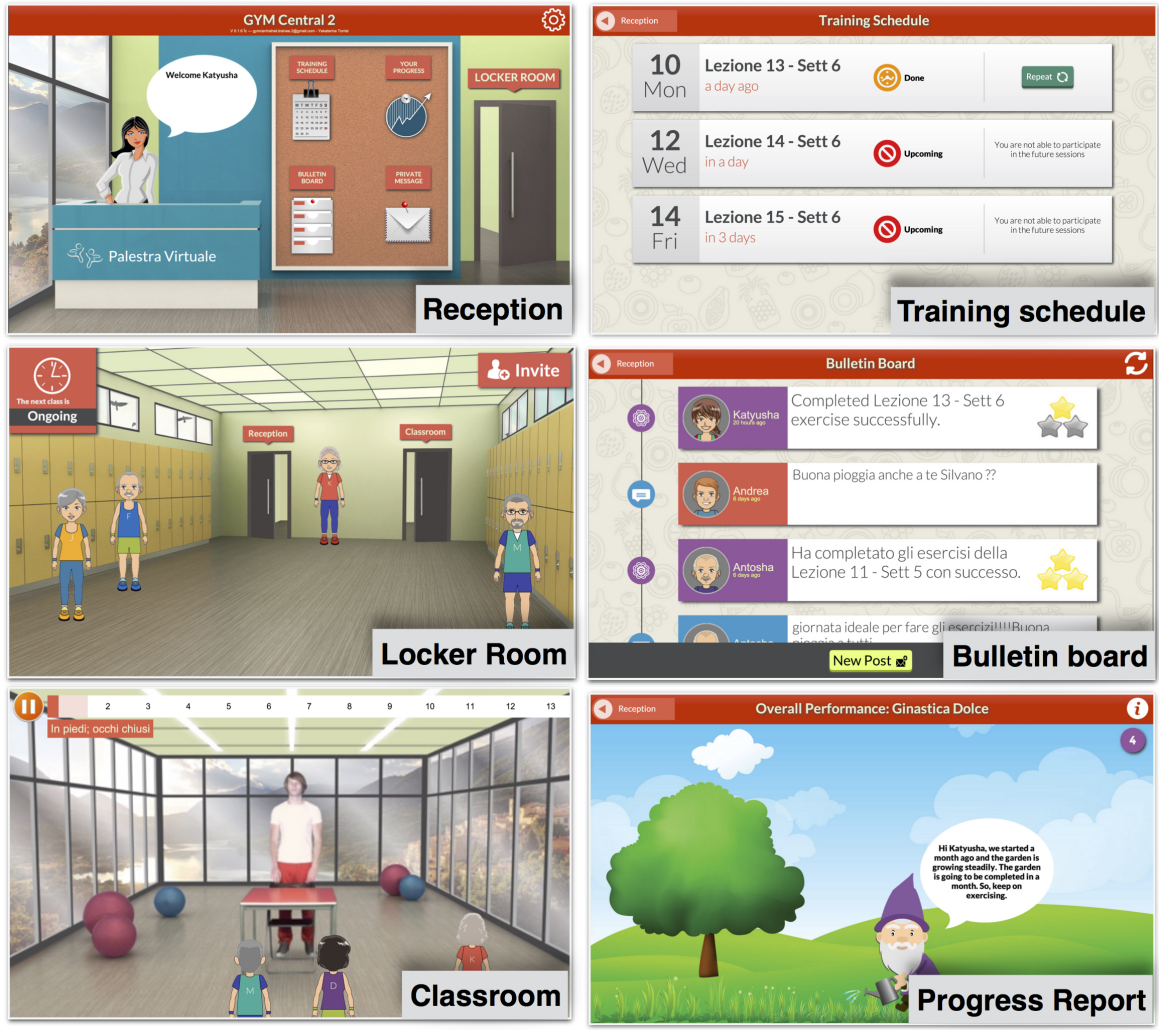
Features of the virtual gym environment of the trainee app.

The technology support was provided by Gymcentral, a tablet and Web app that allows trainees of different functional abilities to follow online group exercises from home, under the supervision of a remote coach [26]. Gymcentral serves the needs of trainees and coach via the *trainee* and *coach* apps (see Figure 1).

The design of the trainee app is based on a virtual gym environment that provides the following main features:

- Tailored training program. It delivers video exercises that are tailored to the abilities and progress of individual trainees. Trainees may receive exercises of different intensity level or not receive some exercises depending on their condition and the coach assessment.
- Online group exercising. It allows trainees to participate in online group exercise sessions in a virtual classroom. Trainees can see the video of the coach and also the presence of other trainees via avatars. However, differences in functional abilities or the intensity level of the exercises remain hidden.
- Persuasion strategies. It provides individual persuasion features such as positive and negative reinforcement and self-monitoring (implemented using a growing garden metaphor), as well as social persuasion features such as social learning, social support, social facilitation, and normative influence.
- Remote monitoring and feedback. Participation to training sessions and completeness of exercises are recorded by the app and made available to the training coach. The coach can act on this data to provide feedback (using the communication features) and increase or tailor the intensity of the training program.
- Communication features. It enables trainees to share public messages with all the other trainees in a bulletin board or to exchange private messages with individual trainees (or the training coach) using an internal messaging feature.

The monitoring and feedback is supported by the coach app, a companion Web app for the training expert.

Details about the features of the Gymcentral app are discussed in detail in the study by Báez et al [26] and the underlying conceptual model in the study by Far et al [27].

### Research Questions

In this work, we studied the feasibility and effectiveness of the online group exercise intervention and its effects on the well-being of Siberian older adults by addressing the following specific research questions (RQ):

RQ1. Is the online group exercising technology usable and accepted by older adults? We aimed at exploring the perception of older adults toward the technology by measuring the usability and acceptance. More importantly, we also explored how the app was used in practice and how the usage relates to the observed effects of the.

RQ2. How do online group exercising and baseline measures influence the adherence of older adults to a training program? Previous research suggests that exercising in a group results in higher adherence and preference by older adults [7,11]. However, research also points to major obstacles when delivering group exercises to heterogeneous populations, which can make training in this setting difficult and less motivating [11]. In this study, we explored how a virtual group environment influences the adherence of older adults under different measures of known determinants of physical activity.

RQ3. Does online group exercising affect the well-being of older adults? We explored the effects of physical training via a virtual social environment on the subjective well-being and social well-being of older adults. By addressing this question, we aimed at contributing to the existing research on the association between physical training and well-being [1-4].

### Study Design

We explored the above questions in two pilot studies in Tomsk, Siberian Federal District (Russian Federation) that adhered to the same protocol and conditions, except for the group cohesion setting:

- Tomsk1 (July 2015-September 2015). Participants with high group cohesion, recruited from two organizations, and with the majority performing shared activities (computer courses and hobbies classes).
- Tomsk2 (April 2016-June 2016). Participants with low group cohesion, recruited from various organizations, with weak or no ties with each other.

As seen above, we explored two group cohesion settings: participants with *strong group cohesion* and participants with *low group cohesion*. Thus, for the reasons explained above, candidate participants from Tomsk 1 had a stronger cohesion than Tomsk 2 at recruitment time, regardless of the treatment they ended up receiving. We did so to understand the effect of the prior connectedness among participants on the observed outcomes.

**Figure 2:**
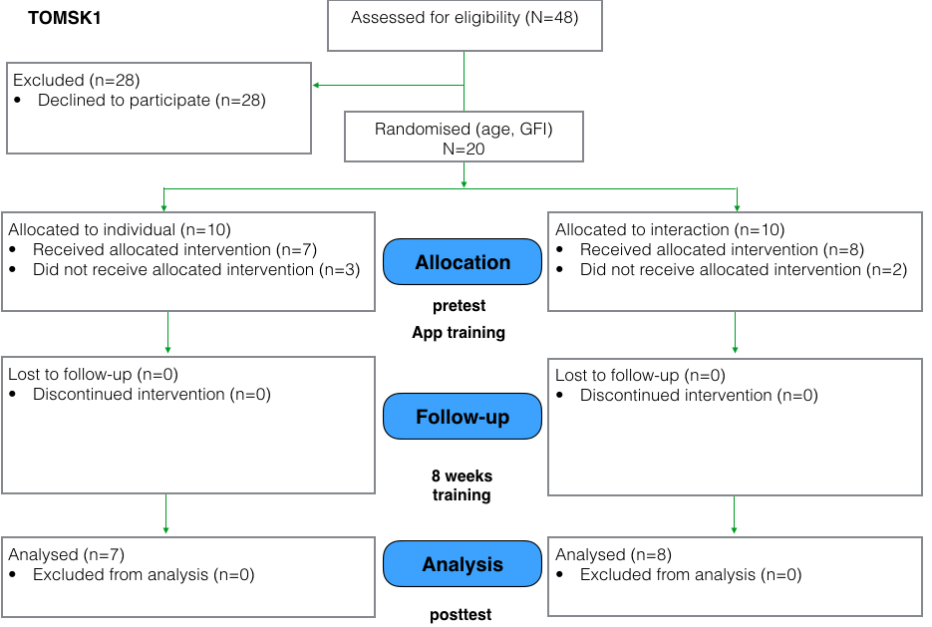
Study flowchart for Tomsk1 (July 2015-September 2015).

**Figure 3:**
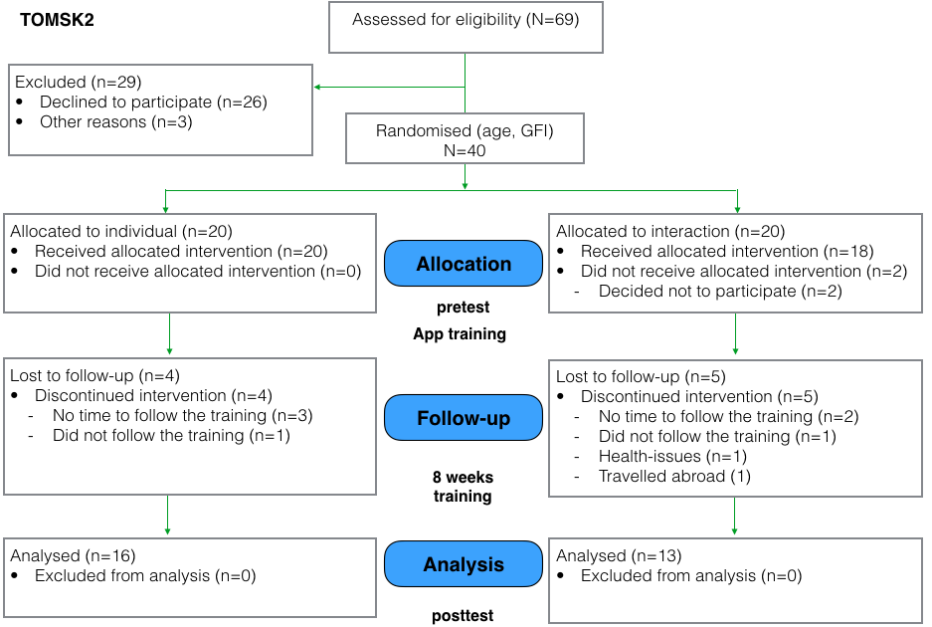
Study flowchart for Tomsk2 (April 2016-June 2016).

Both pilot studies were follow-ups to a previous pilot performed in Trento, Italy, and so they follow the same study design [12]. An overview of the study flow in consolidated standards of reporting trials–compliant format is shown in Figures 2 and 3.

In both studies described here, participants were assigned to an interaction group (online group exercise condition) or to an individual group (individual exercise condition) using a random assignment procedure, with age and participants' frailty level as random assignment variables. In Tomsk 1, the process was slightly different as to ensure a high level of cohesion after randomization: pairs of friends, identified during the informative meeting, were treated as single elements during randomization. In this modified process, we firstly followed the randomization procedure for participants *without friends*, assigning participants to interaction and individual treatments, and then repeating the process for the friend pair units. Thus, friends were assigned to the same treatments, contributing to the overall group cohesion in Tomsk 1.

The two studies and the two treatment conditions defined four effective groups (see Table 1). Participants in the interaction groups have access to online group exercising with social interaction and persuasion features, whereas in the individual groups, participants have access to individual training with persuasion features but with social interactions limited to contacts with the coach. Details about the group cohesion and features available to each group can be seen in Table 1.

**Table 1:** Group cohesion and features of the trainee app available to each study group. Presence of the features in the version of gymcentral application used in each study group are denoted by checkmarks (✓).

Groups		Tomsk 1		Tomsk 2	
		Interaction	Individual	Interaction	Individual
**App features availability**					
	Tailored exercises program (Otago)	✓	✓	✓	✓
	Training with others in the classroom	✓		✓	
	Invitation to join a training session	✓		✓	
	Self-monitoring progress (garden metaphor)	✓	✓	✓	✓
	Positive or negative reinforcement	✓	✓	✓	✓
	Sharing of training activity the in bulletin	✓		✓	
	Contextual messages in the locker room	✓		✓	
	Public messages in the bulletin board	✓		✓	
	Private messages with other trainees	✓		✓	
	Private messages with the coach	✓	✓	✓	✓
**Group cohesion**					
	Weak group cohesion			✓	✓
	Strong group cohesion	✓	✓		

Both versions of the app implemented the same training program, developed on the basis of the Otago exercise program [25], which includes a set of muscle strengthening and balance-retraining exercises. The training program was designed with a standard set of exercises to be performed in each training session, varying in intensity each week according to the performance of the trainees. In the app, each exercise had 10 levels of intensity based on the duration and the number of repetitions. At the beginning of the study, a personal trainer (who was also the coach in the virtual gym) performed a physical assessment, which was used to set the starting intensity level of the program.

Participants received an iPad Air tablet (9.7-inch) preinstalled with the assigned version of the app and Internet access, a case to support the vertical positioning of the tablet, an activity monitoring sensor (Misfit Shine), one pair of ankle weights (0.5 Kg each), and the telephone number of the support team.

Before the start of the training program, participants joined pretest and technology training meetings: (1) an initial meeting where they signed the informed consent and filled out enrollment questionnaires, (2) a session with a medical doctor to evaluate eligibility, (3) a technology training session in the use of tablets and the assigned version of the app, and (4) a session for the physical assessment with the coach and pretest measures. The technology training followed a workshop format and was done in small groups of 10 participants each. Participants assigned to individual and interaction conditions attended workshops separately as they were provided with different versions of the app.

In the 8 weeks of the training, participants performed the home-based training activity with the monitoring of the coach and of the support staff. The training schedule offered three exercise sessions per week, and participants were required to perform at least two exercise sessions every week. The duration of the training session ranged from 30 to 40 min depending on the intensity level. Participants were free to join the training sessions at any time. Posttest measures took place on the week after the training.

The coach guiding the participants during the training was a practicing doctor with a primary care doctor degree and had over 10 years of experience in gymnastics, rehabilitation exercises, and yoga for older adults. Before the beginning of experiment, the coach was acquainted with the Otago training program and Gymcentral app settings.

During the training period, the coach had the task of progressing the intensity of the exercise program and providing feedback. At the end of every week, the coach could maintain or increase the intensity level of each trainee according to the attendance and completeness of the training sessions in the week. The coach was also instructed to contact trainees at least once a week to provide feedback and to respond to any question from the trainees. The coach was not aware of the difference between the interaction and individual groups, and both received the same amount of technical support.

The pretest measures included the *Groningen Frailty Indicator* [28], the *Rapid Assessment of Physical Activity Questionnaire* [29], demographic information, and questionnaires concerning psychological and social well-being. The posttest measures included the *System Usability Scale* (SUS) [30], a set of questions on the acceptance of the app, the *Satisfaction with Life Scale* (SWLS) [31,32], the medical outcomes survey (*MOS) Social Support Scale* [33,34], and the 3-item *revised University of California, Los Angeles (UCLA) Loneliness Scale* (R-UCLA Loneliness Scale) [35,36]. The participants filled in all the questionnaires by themselves in pencil-and-paper format.

The study protocol received ethical approval from the CREATE-NET Ethics Committee on ICT Research Involving Human Beings (Application N. 2014-001) in Trento, Italy. The studies reported in this paper—as follow-ups to our previous study—comply with this protocol, with the informed consent and informational materials translated into the Russian language.

### Participants

We considered eligible for the study: participants aged 59 years or older, independent living, self-sufficient, and with a nonfrail, transitionally frail or a mild frailty level. These criteria were measured by self-reports. All participants had to pass a doctor assessment to ascertain the absence of conditions that would prevent them from performing light physical exercises. Participants wearing pacemakers were considered not eligible as the study required the use of an activity sensor (Misfit shine monitor). The specifics of baseline measures for each study site are described in Table 2.

**Table 2:** Baseline measures for study site. Tomsk1 and Tomsk2.

Measures			Individual	Interaction	*P* value^a^
**Pre allocation test**					
	**Age (years), mean (SD)**				
		Tomsk1	65.0 (6.1)	68.2 (7.8)	.71
		Tomsk2	68.8 (7.2)	67.6 (6.2)	.48
	**Females, n (%)**				
		Tomsk1	100 (100)	90 (90)	
		Tomsk2	100 (100)	100 (100)	
	**Groningen frailty indicator, mean (SD)**				
		Tomsk1	4.2 (2.04)	4.5 (2.42)	.99
		Tomsk2	3.6 (2.54)	3.56 (2.5)	.91
	**Rapid Assessment of Physical Activity Questionnaire, mean (SD)**				
		Tomsk1	5.78(1.79)	5.9 (1.73)	.72
		Tomsk2	5.15(2.41)	5.13(1.96)	.84
**Post allocation tests—Self-reported**					
	**Physical Activity Enjoyment Scale, enjoyment, mean (SD)**				
		Tomsk1	50.0 (3.5)	50.0 (4.8)	.99
		Tomsk2	49.9 (5.4)	47.8 (4.2)	.49
	**R-UCLA**^b^**Loneliness Scale, loneliness, mean (SD)**				
		Tomsk1	4.2 (1.6)	5.4 (1.4)	.18
		Tomsk2	4.3 (1.1)	4.0 (1.2)	.35
	**MOS**^c^**Social Support Scale, social support, mean (SD)**				
		Tomsk1	4.0 (1.5)	5.1 (1.6)	.99
		Tomsk2	4.3 (1.1)	4.0 (1.2)	.55
	**SWLS**^d^**, well-being, mean (SD)**				
		Tomsk1	4.0 (1.5)	5.4 (1.4)	.52
		Tomsk2	4.3 (1.1)	4.1 (1.2)	.35
**Post allocation tests—Physical assessment**					
	**Leg muscle strength, mean (SD)**				
		Tomsk1	13.6 (2.2)	12.9 (1.4)	.49
		Tomsk2	16.5 (3.8)	16.5 (3.0)	.96

^a^Differences computed using independent samples *t* test for age and leg muscle strength; all the other variables were analyzed with Mann Whitney tests.

^b^R-UCLA: revised-University of California, Los Angeles.

^c^MOS: Medical Outcomes Survey.

^d^SWLS: Satisfaction with Life Scale.

Participants in both studies were contacted through retirement organizations in Tomsk, Russia. In the first study, Tomsk1, participants were mainly invited through organization offering computer-learning classes and hobbies activities for seniors. In the Tomsk2 study, the recruitment was carried out through three organizations organizing social activities and events. We conducted presentations explaining the project and their expected involvement and handed out printed bulletins. Older adults interested in participating provided their phone numbers and were later on contacted by the project coordinator. Details about the retirement organizations and the number of candidates reached can be seen in Table 3.

In the Tomsk1 study, 20 participants were found eligible for the study (mean age individual group=65, SD 6.1; interaction group: mean 68.2, SD 7.8; 19 females and 1 male). In the Tomsk2 study, 40 participants were accepted according to the inclusion criteria (mean age individual group=68.9, SD 7.2; interaction group: mean 67.6, SD 6.2; all 40 female). The difference in the number of male and female participants is because of the demographics of the study location and the availability of male candidates at the retirement organizations. In Siberia, lifespan gap between males and females is one of the biggest in the world: life expectancy at birth for men is 64.7 years, whereas for women it is 76.3 years [37]. These demographics posed difficulties in recruiting male participants from the retirement organizations. The study flow for Tomsk1 and Tomsk2 is depicted in Figures 2 and 3. After the recruitment, participants in both studies signed the informed consent before participating in the experiment.

In the Tomsk 1 study, out of 20 participants, 5 withdrew before the start of the study for health problems or personal reasons; therefore, data of 15 participants was included in the analysis. In the Tomsk2 study, out of 40 participants, 2 withdrew before the beginning of the training because of travel plans. During the training program, 4 participants in the individual group and 5 participants in the interaction group dropped out because of health issues, travels, or reported lack of time for participation. Thus, in the Tomsk2 study, a total of 29 participants were included in the analysis (individual: 16, interaction: 13).

There were no statistical differences between individual and interaction groups in term of initial measures (Table 2). These baseline comparisons have been performed on participants that finished the training program.

### Outcome Measures

#### Acceptance and Usability

We focus on the usability, acceptance of the technology, and preference to train together:

- Usability: The usability of the app was evaluated by means of the SUS [30]. This scale includes 10 items rated on a 5-point Likert scale (from 1=“completely disagree” to 5 =“completely agree”). The SUS score ranges from 0 (low usability) to 100 (high usability). However, in a pretest of the scale, older adults found difficult to understand two items (“I found the various functions in this system were well integrated” and “thought there was too much inconsistency in this system.” Therefore, we decided to exclude these two items in the questionnaire we administered to our participants. This means that the SUS score in our study ranged from 0 to 80.
- Acceptance: Acceptance was measured with a set of questions designed to evaluate positive (“I enjoy using the app”) or negative feelings (“The app makes me nervous”) associated with the use of the apps, the response to the communication feature (“It is easy to communicate with other people with the app”), the intention to use it (“I would like to use the app in the future”), and the perceived ease of use (“It is easy to use the virtual gym to perform exercises”). These questions were rated on a 5-point Likert scale (from 1=”completely disagree” to 5=”completely agree”). The questionnaire was developed by our team on the basis of previous literature [38]. Each question has been separately analyzed.
- Copresence: Participants had the choice to train at any time, but they could also coordinate to train at the same time via texting or using the *invite user to join* feature. To capture the preference of users for group training, we logged the attendance to the training sessions to compute for each user whether he or she trained alone (individual attendance) or together with another trainee (joint attendance). We then define copresence of a group as the ratio of joint attendances with respect to the total number of attendances.

**Table 3:** Senior citizen organizations contacted and candidates reached in each study.

Retirement organization	Study	Size of groups reached
Tomsk union of retirees	Tomsk 1	Large organization providing courses to around 600 retirees per year. Four active courses at the time (approximately 20 members each) were contacted, reaching around 80 older adults in total
Veterans council of Tomsk Polytechnic University (TPU)	Tomsk 1	Small organization of around 80 retirees. The invitation was extended to all members
Veterans Council of Tomsk Scientific Center	Tomsk 2	Small organization of around 80 retirees. The invitation was extended to all members
Tomsk region veterans council	Tomsk 2	Small organization of around 100 retirees. The invitation was extended to all members
Veterans council of TPU	Tomsk 2	Small organization of around 80 retirees. The invitation was extended to all members

#### Adherence to the Training

Measured with:

Persistence: Persistence was computed considering the ratio between the number of attendances to exercise sessions by a participant and the number of the exercise sessions planned in the program. Participation was measured by logging the attendance to the scheduled training sessions in the virtual classroom. For persistence, a rate equal to 100% was considered as participation in all three sessions per week, for all 8 weeks of training. Participants were not aware of how the persistence was scored but could monitor the individual progress in the garden (self-monitoring feature).

#### Subjective Well-Being, Social Support, and Loneliness

To measure if there was an improvement in the well-being outcomes as a result of training (secondary outcomes), we relied on the following instruments:

- SWLS [31]: Five questions rated on a 7-point Likert scale (from 1=”Strongly disagree” to 7=”Strongly agree”). The SWLS was translated and adapted to the Russian language by Tucker et al [32]. The total score ranges from 5 to 35, with higher scores indicating higher levels of life satisfaction.
- Loneliness: To measure loneliness, we used a shorter version of the R-UCLA Loneliness Scale [35] developed by Hughes et al [36]. The scale used includes 3 items scored on a 5-point Likert scale, with the total score ranging from 3 to 15 and higher scores indicating higher levels of loneliness.

#### Determinants of Physical Activity

In the analyses explained in the following sections, we use the following determinants of physical activity as covariates:

- Physical Activity Enjoyment Scale (PACES) [39]: This scale includes 16 items scored on a 5-point Likert scale (from 1=“disagree a lot” to 5=“agree a lot”). The PACES total score ranges from 16 to 80 (maximum enjoyment).
- MOS Social Support: [33,34]: Eight questions scored on a 5-point Likert scale (from 1=“None of the time” to 5=“All of the time”). This scale was translated by us according to the international guidelines [40]. It aims at measuring the social support provided by others. The total score ranges from 1 to 8, with higher scores indicating higher levels of social support.
- Leg muscle strength: Measured with the 30-second chair stand test [41]. The purpose of this test is to evaluate leg strength and endurance. From a seated position, the participant rises to a full standing position and then sits back down again for 30 seconds. The outcome measure is the number of times the participant comes to a full standing position in 30 seconds.

### Statistical Analysis

We analyzed the difference between the interaction and the individual groups in terms of the SUS score with two Mann Whitney tests, whereas for the difference in the percentage of copresence, we use *t* tests.

We analyzed adherence (measured as rate of persistence) to the training program with an analysis of covariance (ANCOVA) with group (interaction vs individual) and study (Tomsk1 vs Tomsk2) as between-subject factors and leg muscle strength, social support (MOS score), and enjoyment of physical activity (PACES score) as covariates.

For well-being measures, we selected the SWLS score and R-UCLA Loneliness Scale score as dependent variables to be used in two separate repeated-measures analysis of variance (ANOVA). We used the same independent variables in both ANOVAs: time (pretest vs posttest) as within-subject factor and group (interaction vs individual) and study (Tomsk1 vs Tomsk2) as between-subject factors.

The statistical analyses were performed using the open source statistical software R (R Studio Team) [42], using the ggplot2 package to create plots [43].

## Results

### Perception and Adoption of the Technology

A starting point to understand the feasibility of the technology for our target population was to address (RQ1) and investigate the perceived usability, acceptance, and usage of the online group exercising technology.

#### Usability

Nine participants did not answer to some of the questions of the SUS and thus have been excluded by the analysis on this account. On average, the SUS score (on an 80 points scale, as we excluded two questions) was very similar between the interaction group (mean 63 [SD 9]; N=19; range 48-80) and the individual group (mean 66 [SD 14]; N=15; range 40-80). From a more detailed perspective, a Mann Whitney test showed that neither in the Tomsk1 study (W=11, *P* ≥.99) nor in the Tomsk2 study (W=89.5, *P*=.32) the SUS scores were different between the two groups (individual vs interaction) despite the higher complexity of the app assigned to the interaction groups.

#### Acceptance

Table 4 reports the results for the questions concerning acceptance (A). Consistently with the SUS score, trainees showed a high level of acceptance of the app. In fact, as the Table shows, trainees reported high levels of enjoyment (A1) and low levels of nervousness (A2) in using the app. Training with the app was perceived as very easy to do (A4) as well as communicating (A3), but with a lower score by 1 point. Trainees also reported with a high score their intention to use the app in the future.

#### Characterization of App Usage

**Figure 4:**
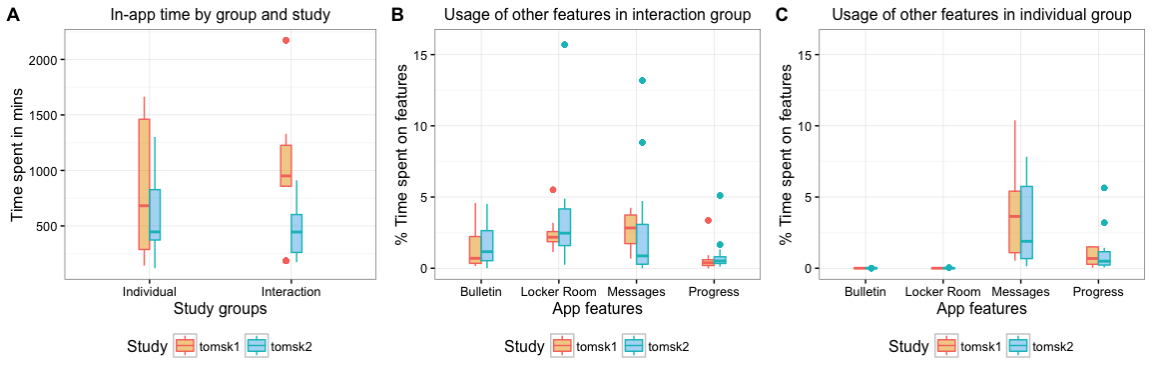
App usage by group and study. (A) Total time (in min) spent by user in the app during the experiment, (B) Usage of the app features in the interaction group, and (C) Usage of the app features in the individual group, % from total time spent in the app.

To characterize the usage of the various features of the app, we analyzed the app logs to derive how participants spent their time in the app. Overall, the mean time spent in-app was higher in Tomsk1 (16 hours) compared with Tomsk2 (9 hours), the difference being marked by a higher time spent by the interaction group in the first study (see Figure 4).

**Table 4:** Mean (SD) of the technology acceptance (A) responses for each group and study (range:1-5).

Features	Tomsk1		Tomsk2		
	Interaction, mean (SD)	Individual, mean (SD)		Interaction, mean (SD)	Individual, mean (SD)
A1 (feel joy)	3.9 (1.4)	3.9 (1.6)		2.8 (1.9)	3.3 (1.9)
A2 (feel nervous)	2.3 (1.2)	1.2 (0.4)		1.4 (0.8)	1.1 (0.3)
A3 (easy social)	4.4 (0.9)	3.0 (2.3)		3.1 (1.7)	4.1 (1.5)
A4 (easy train)	4.9 (0.4)	4.6 (1.1)		4.7 (0.5)	5.0 (0)
A5 (future use)	4.9 (0.4)	4.2 (1.8)		4.6 (0.7)	5.0 (0)

Not surprisingly, most of the time was spent training in the classroom, as the duration of exercise session ranged from 20 to 40 min depending on the intensity level. Looking at the time spent in the classroom relative to the time spent in-app by each participant, we can see that participants of the individual group in both studies spent nearly the same percentage of their time (Tomsk1=95.3%, Tomsk2=95.6%) in the classroom. Participants in the interaction groups spent a little less on the classroom—especially in Tomsk2 (Tomsk1=92.5%, Tomsk2=81.4%). The lower use in the interaction app is because of the presence of extra features and in the case of Tomsk2, because of the lower time spent training.

Analyzing the usage of the other features, we observe that participants spent a significant percentage of their time messaging, particularly those in the individual groups (see Figure 4). We can derive that the individual group not only used the training feature but also the messaging tool to interact with the coach and to check their progress. The bulletin board and the locker room were not available for the individual group.

The interaction group also used the social features (see Figure 4). The messaging feature was used to send private messages to other participants and the coach, especially in Tomsk1. The bulletin board was also used, although visits were more related to a *lurking* behavior rather than actual contributions. We attribute this to automatic sharing of the participant’s performance (as a 3-star rating based on completeness) on the bulletin board (*social learning* persuasion strategy [12]). The locker room comprises also an important percentage but it is mostly because of the fact that it preceded the classroom in the navigation. No important interactions or invitation to the join the classroom were registered from this virtual space.

#### Online Interactions

Participants in the interaction group had the possibility of exchanging public and private messages either with the coach or other trainees, whereas in the individual group, the interactions were limited to private messages with the coach. Table 5 summarizes the exchanges among participants of both groups in the two pilot studies.

Participants in the social condition made significantly more use of private messages compared with public messages. This was the case even for participants in Tomsk1 (strong group cohesion), with 4.4 private messages compared with only 0.6 public messages per user. Not surprisingly, participants of Tomsk1 interacted significantly more among themselves (4.4 messages per user compared with only 0.4 in Tomsk2).

It is also noteworthy the asymmetry between sent and received messages when including messages by the coach. This is because of the scheduled messages by the coach who reached participants on a weekly basis but was not always reciprocated, as well as to the interaction behavior of the coach, that is, sending more than one messages per interaction.

#### Copresence in the Training

**Figure 5:**
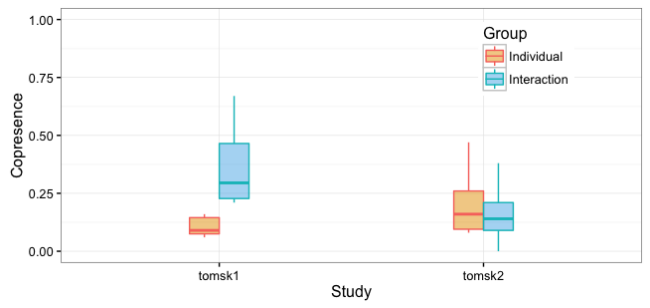
Copresence by study and group.

Participants in the interaction group were able to see each other, train together, and coordinate their participations. Participants in the individual group were not. Thus, copresence in the individual group is only an indication of meetings by chance and used for comparisons. The copresence by study and group is shown in Figure 5.

The copresence in the Tomsk1 study was on average significantly higher in the interaction group: 36.25% (SD 17.25%) in comparison with 10.71% (SD 4.15%) for the individual group. A *t* test showed a significant difference between the interaction and individual groups (*t*_7.9_=−4.05, *P*=.004) in favor of the group training condition.

In the Tomsk2 study, the copresence was of 16.38% (SD 11.44%) in average in the interaction group and 19.4% (SD 11.13%) in the individual group. A *t* test showed no significant difference between groups (*t*_25.22_=0.7, *P*=.49).

### Program Adherence

The overall persistence rate was of 74% (SD 27%) when considering the number of sessions available in the 8 weeks of training. Breaking down this number by group treatment, we observe a persistence rate of 75% (SD 28%) for the individual groups and 74% (SD 26%) for the interaction groups, whereas the result by study shows a persistence rate of 82% (SD 24%) for Tomsk1 and 70% (SD 28%) for Tomsk2. In the study Tomsk1, the persistence rate was 77% (SD 25%) for the individual group and 87% (SD 23%) for the interaction group; in Tomsk2, it was 74% (SD 30%) for the individual group and 65% (SD 25%) for the interaction group.

An ANCOVA was performed to compare the persistence of participants of individual and interaction groups in the two studies while controlling for the initial baseline measures of leg muscle strength, social support, and PACES. The results show neither a significant main effect for group (*F*_1,18_<1, *P*=.74) or for study (*F*_1,18_=1.46, *P*=.24), nor interaction between study and group (*F*_1,18_=1.15, *P*=.30).

**Figure 6:**
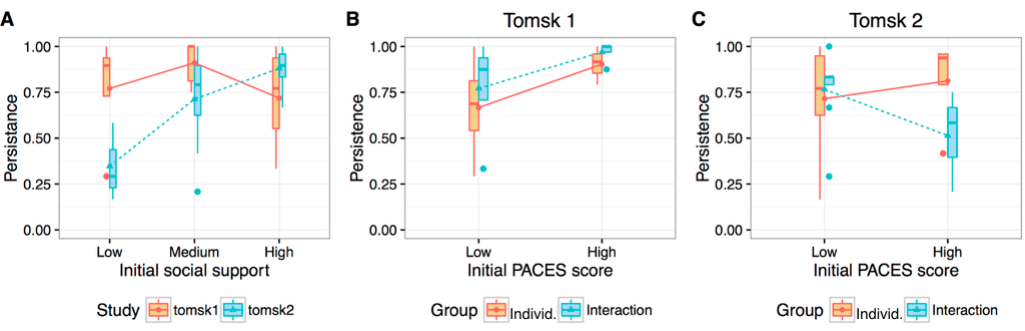
Interaction plots for persistence and baseline measures. (A) Interaction between study and initial level of social support (medical outcomes survey, MOS score has been grouped in three equally distributed intervals: low, medium, and high). (B) Interaction between group and initial PACES score in Tomsk1. (C) Interaction between group and initial PACES score in Tomsk2.

Considering the baseline measures, the results show a significant interaction between study and the initial social support score (*F*_1,18_=5.23, *P*=.03). As observed in Figure 6, part A, in Tomsk2, participants with higher social support level showed higher adherence to the training, whereas in Tomsk1, the adherence is not significantly associated with by the initial social support score.

**Table 5:** Mean (SD) messages exchanged among all users (including the coach) and only trainees.

Messages exchanged		Tomsk1		Tomsk2	
		Interaction, mean (SD)	Individual, mean (SD)	Interaction, mean (SD)		Individual, mean (SD)
**Private messages sent**						
	All users	8.4 (6)	8.1 (7)	4.3 (6)		5.7 (4)
	Only trainees	4.4 (3)	Not applicable (N/A)	0.4 (1)		N/A
**Private messages received**						
	All users	13.5 (2)	13.1 (7)	11.1 (3)		10.9 (1)
	Only trainees	4.3 (2)	N/A	0.5 (1)		N/A
**Public messages posted**						
	Trainees	0.6 (1)	N/A	0.5 (1)		N/A

No significant effects were found for the initial scores of leg muscle strength.

The interaction between PACES score and group was also significant (*F*_1,18_=6.001, *P*=.03). As shown in Figure 6, in Tomsk2, participants with higher enjoyment of physical activity had a higher adherence level (Figure 6, part B), whereas in Tomsk1, enjoyment of physical activity had a negative effect on the interaction group (Figure 6, part C).

### Well-Being Outcomes

Eight participants did not answer to one or more questions of the SWLS and thus, have been excluded by this analysis. On the subset of participants without missing answers, SWLS score was analyzed with a repeated measure ANOVA with time (pretest vs posttest) as within-subject factor, and group (individual vs interaction) and study (Tomsk1 vs Tomsk2) as between-subject factors. Only the main effect of time was significant (*F*_1,31_=5.85, *P*=.02). Participants reported high satisfaction in the posttest questionnaire (mean 23.8 [SD 6.2]) compared with the pretest measures (mean 21.34 [SD 5.8]).

The same analysis was performed on R-UCLA Loneliness Scale. Eight participants were excluded from the analysis because of missing values in the pretest or posttest questionnaires. Only the main effect of study showed a tendency toward significance (*F*_1,31_=3.55, *P*=.07). Participants reported a lower level of loneliness in the Tomsk1 study (mean 4.77 [SD 1.7]) compared with the Tomsk2 study (mean 4 [SD 1]).

## Discussion

### Principal Findings

#### Online Group-Exercising Tool Rated as Highly Usable (Research Question 1)

Participants’ rating on the usability of the app shows that the group exercise app (assigned to the interaction group) has a high usability and that the added complexity in relation to the more traditional home-based version (assigned to the individual group) did not significantly affect its usability.

When asked in detail, participants reported the training feature as very usable, whereas the messaging as usable but with a lower score (1 point lower), possibly because of the typing. The intention to use the app in the future was also very high, which along with the analysis of the actual usage, points to the feasibility of using the online group-exercising tool for training in a social context. These results are in line with a previous usability study and usage behavior analysis done on the Gymcentral tool [26].

#### Private Messages as Preferred Interaction Channel Among Trainees, Even in the Strong Cohesion Group (Research Question 1)

As in our previous study analyzing online interactions in a training context among Italian older adults [26], we expected to observe a higher usage of public messages for communication among trainees. Surprisingly, however, participants exchanged more private messages among themselves than public ones, even in the strong cohesion group. The high cohesion setting only accounted for more exchanges per user, not for group-level interactions. This result suggests different attitudes toward group interactions possibly because of cultural differences. In fact, the usage logs suggest mainly a lurking behavior, possibly because of the automatic sharing of the participant’s performance—a social learning feature. Thus, further studies are required to design better online interaction tools that would motivate group building in the cultural context of reference.

#### Copresence Higher in the Strong Cohesion Group (Research Question 1)

The results of copresence show us that participants from the interaction group in Tomsk1 (strong cohesion group) participated in significantly more training sessions with the company of others compared with the meetings by chance in the individual group. We have seen the same effect in our previous study [12] featuring a high-cohesion group of Italian older adults. This effect was not observed in Tomsk2 (low cohesion group), suggesting that training together is not necessarily a preference in groups with low cohesion, and thus, the cohesion level might affect the willingness to train together.

#### Online Group Exercising Did Not Result in Higher Adherence When Compared With Individual Training With Persuasion Features (Research Question 2)

We have observed a higher adherence for the groups with high cohesion, and in particular, under the group-exercising treatment (interaction: mean 87% [SD 23%]; individual: mean 77% [SD 25%]). However, the ANCOVA showed neither a significant main effect for group or for study, nor interaction between study and group. This suggests that the added group exercising feature did not account for a significant difference in persistence rate compared with the individual training with persuasion features (interaction: mean 65% [SD 25%]; individual: mean 74% [SD 30%]).

In our previous study with Italian older adults [12], we observed a higher adherence to the online group-exercising compared with individual training (with no persuasion strategies). Here, we did not observe the same effect when comparing online group exercising with individual training (with persuasion strategies). We attribute this effect to (1) Persuasion features in the individual training condition that raised the adherence by 10% compared with our previous study [12]. This increase made the difference in favor of the group exercising condition nonsignificant and (2) Weaker cohesion among participants in Tomsk2, which might have reduced the effect of normative influence and peer support, resulting in a 20% drop in adherence compared with Tomsk1 and our previous study [12].

These results contribute to the ongoing discussion on the differences between individual and group training (see [21] for the most recent meta-analysis on the topic). First, it adds to the evidence that group-exercising in low cohesion groups results in an adherence comparable to that of individual training with contact (with a coach), extending the evidence to online settings. Second, it partially supports the evidence that group exercising in high-cohesion groups results in higher adherence than individual training with contact. On this point, we have seen evidence only when comparing group exercising with individual training with no persuasion strategies, which is indeed closer to the individual condition explored in [21]. The possibility of incorporating persuasion strategies in online setting adds a new dimension that requires further investigation.

#### Social Support Can Predict Adherence to a Training Program When Social Connections are Weak or Absent (Research Question 2)

In analyzing the effects of social support on adherence, we have seen a significant interaction between study and the initial social support score at baseline. In Tomsk2, participants with higher social support level showed higher adherence to the training. This suggests that higher level of social support is associated with higher levels of adherence when the connection among participants is weak (Tomsk2). This observation is in lines with the literature highlighting the social support structure as an important determinant of adherence [7,8]. Interestingly, Tomsk1 did not show a significant association between initial social support and adherence. This suggests that low levels of external social support (as measured at baseline) can also be compensated with the social dynamics of an online group with strong cohesion (Tomsk1).

#### Enjoyment of Physical Activity With Contradicting Effects on Adherence for Groups With Weak and Strong Cohesion (Research Question 2)

Enjoyment of physical activity is described as determinant of physical activity [7,8] and is associated with positive attitudes toward exercise, intrinsic motivation, and consequently long-lasting adherence to physical activity [44,45]. We have seen, however, some conflicting effects of this variable—as measured with the PACES scale—on the adherence of the groups with weak and strong cohesion: all groups showed higher adherence for higher PACES score except for the interaction group with low cohesion that showed the opposite effect. This negative effect on adherence in the latter group came as a surprise, and it requires further study to investigate its roots and whether it is because of negative social dynamics in low cohesion settings.

#### Initial Level of Fitness With Nonsignificant Effect on Adherence of Online Group Exercising and Individual Training With Persuasion Strategies (Research Question 2)

Implementing group exercising can be challenging, especially in heterogeneous populations. Individual differences among older adults can lead to motivational issues and problems in tailoring the training [11]. In addition, perceived barriers such as lack of skills, pain, fear of injuries, and falls can also constitute obstacles to the motivation of older adults to exercise.

In our previous study with Italian older adults [26], we observed that the initial level of fitness could predict the adherence of older adults to an individual training (without persuasion strategies). It was also observed that the online group exercising tool—the same used in the pilots reported in this paper—was effective in mitigating that effect. In lines with this prior study, the results from our two pilots showed that the initial level of fitness did not have a significant effect on adherence of the interaction group but neither on the adherence of the individual group. One potential explanation is the presence of individual persuasion strategies in the version of the app used by the individual group, which might have leveled the effect. This suggests that more studies are needed to better understand the roots of the observed effects of the initial level of fitness, as well as the effects of individual and social persuasion in mitigating them.

#### Seasonal Fluctuations and Its Influence on Availability of Candidate Participants (Research Question 2)

Seasonal fluctuation has been found to determine the level of physical and social activities of older adults [13], especially in high latitudes where winter can result in a decline of physical functions of older adults [14]. In Siberia, these fluctuations greatly influence the activities of the daily living and the opportunities to engage in activities in general.

Although our studies were set in spring and summer periods, we did experiment the effects of the seasonal fluctuation but at recruitment and for quite the opposite reasons. June to September is gardening season, and independent living older adults usually engage in this activity, spending most of the period in their summer houses (Dacha). This influenced the availability of participants in our study as it created obstacles for some candidates that showed initial interest in participating (eg, finding time to train and worries of bringing tablets with them outdoors or to the Dacha). After this experience, the second study was moved to earlier spring months (April-June) to increase the pool of potential candidates. However, we did not see a significant difference in the program adherence that could be explained by these two different seasons. Further studies are needed, especially to understand the effects of the extreme winter season.

#### Increase in Life Satisfaction as a Result of the Training, Regardless of the Version of the App (Research Question 3)

Recent history, along with current social, political, and economical factors have impacted negatively in life satisfaction and happiness of older adults in the Russian Federation [17]. Thus, devising and studying solutions aiming increasing the happiness and well-being of older adults in this region is of paramount importance.

In investigating the impact of physical training, we have seen an overall improvement in the SWLS score for all participants, regardless of the version of the tool used. This is consistent with our previous study with Italian older adults [26], where we observed an improvement in the subjective well-being of the participants regardless of being part of the individual or group condition. Furthermore, these results are in line with previous literature on the benefits of physical activity on the QoL of older adults [46,47], and contribute with additional evidence in favor of technology-supported interventions and their benefit for older adults in the Siberian region.

#### No Significant Decrease in Loneliness, Despite Social Features (Research Question 3)

Participants did not observe any decrease in the loneliness score as a result of the training, not even those in the online group exercise condition. This is contrary to our expectations, given the social context provided by the group-exercising and the social interaction features. In Trento, Italy [26], we did observe a significant decrease in the loneliness score, but compared with this study, the usage of social interaction tools and adherence to the training was much higher. This difference in the usage of social interaction features, possibly because of cultural differences as reported earlier, could have limited the effectiveness of the medium.

### Limitations

#### Gender Imbalance

The lifespan gap between males and females in the Siberian region is one of the biggest in the world: life expectancy at birth for men is 64.7 years, whereas for women it is 76.3 years [37]. These demographics limit the availability of male candidates in the senior citizen organizations, and therefore, our ability to recruit more male participants. However, previous studies suggest that male and female participants may have the same reactions to sport activities despite differences in motives to participation [45,48]. Still, further studies are needed to see if these observations can be translated to the intervention described in this paper.

#### Group Size Difference

The amount of participants in the Tomsk2 study was twice bigger than in the first Tomsk1 study, 40 and 20 participants, respectively.

The difference in the group size between the two studies is because of (1) the complexity of the study design and (2) the difficulty in finding participants of older age willing to participate, given the specific social characteristics of the region (older adults living in Siberia are not used to participate in studies). Therefore, we were able to involve only 20 participants for the study Tomsk 1. The following year, as we built better contacts with various retirement organizations and local organizations became more familiar with the project, we were able to involve 40 people in the study (Tomsk 2).

#### No Quantitative Measures of Group Cohesion

Group cohesion was defined as a property of the pool of candidates: participants acquainted with each other and engaging in joint activities. This property was maintained during randomization by ensuring that pairs of friends would end up in the same groups. While being a solid definition, the fact that cohesion was not qualitatively measured should be noted as a limitation.

#### Scales Validation in Russian Language

There is a lack of translations of international standardized measure in Russia. Therefore, except the SWLS (which has already been validate in Russian language), no translation was available for the measures used in the study. These measures were translated and adapted to Russian language and culture by our research group by using the standard translation or bask-translation procedure. During this procedure, we ensured to reach semantic, idiomatic, and conceptual equivalence between the original English and final Russian versions.

Although, without a validation study, we cannot be completely sure that these instruments fully fit the socioeconomic characteristics of Siberia, we believe that the standard procedure adopted to translate these instruments provided reliable results. This should be considered as the limitation of the study.

#### Validity of the System Usability Scale

Two questions were excluded from the SUS because in the pretest of the prefinal version of the scale (during the translation or back-translation procedure), older adults found it difficult to understand them (“I found the various functions in this system were well integrated” and “I thought there was too much inconsistency in this system”). Therefore, whereas in the original scale the total SUS score ranged from 0 to 100, in our study it ranged from 0 to 80. This is a limitation of our study and could make it difficult to interpret the usability results. However, it is worth noting that no usability scale suitable for older adults existed in Russian language, and our study provides the first adaptation for this culture. Future studies should investigate the validity of this short version of the SUS.

### Conclusions

The results point to the feasibility and effectiveness of technology-supported physical interventions, and in particular, of online group exercising among Siberian older adults. High cohesion groups are preferable for group exercising, especially to mitigate effects of low social support on adherence. Cultural differences might explain the preference of private messages over public ones. Results in terms of subjective well-being are promising, but enabling interaction has proved not to be enough to observe a decrease in loneliness. Thus, further research is needed to understand how to better enable community-building interactions.

## Abbreviations

ANCOVA: analysis of covariance
ANOVA: analysis of variance
MOS: medical outcomes survey
PACES: Physical Activity Enjoyment Scale
QoL: quality of life
RQ: research question
R-ULCA: revised University of California, Los Angeles
SUS: System Usability Scale
SWLS: Satisfaction with Life Scale
